# Conceptualizing socially-assistive robots as a digital therapeutic tool in healthcare

**DOI:** 10.3389/fdgth.2023.1208350

**Published:** 2023-07-14

**Authors:** Cedomir Stanojevic, Casey C. Bennett, Selma Sabanovic, Sawyer Collins, Kenna Baugus Henkel, Zachary Henkel, Jennifer A. Piatt

**Affiliations:** ^1^ Department of Parks Recreation and Tourism Management, College of Behavioral and Social Health Sciences, Clemson University, Clemson, SC, United States; ^2^ Department of Intelligence Computing, Hanyang University, Seoul, Republic of Korea; ^3^ Department of Computing & Digital Media, DePaul University, Chicago, IL, United States; ^4^ School of Informatics and Computing, Indiana University, Bloomington, IN, United States; ^5^ Department of Computer Science and Engineering, Mississippi State University, Mississippi State, MS, United States; ^6^ Department of Health and Wellness Design, School of Public Health, Indiana University, Bloomington, IN, United States

**Keywords:** digital health, digital therapeutics, socially assistive robots, artificial intelligence, machine learning, internet of things

## Abstract

Artificial Intelligence (AI)-driven Digital Health (DH) systems are poised to play a critical role in the future of healthcare. In 2021, $57.2 billion was invested in DH systems around the world, recognizing the promise this concept holds for aiding in delivery and care management. DH systems traditionally include a blend of various technologies, AI, and physiological biomarkers and have shown a potential to provide support for individuals with various health conditions. Digital therapeutics (DTx) is a more specific set of technology-enabled interventions within the broader DH sphere intended to produce a measurable therapeutic effect. DTx tools can empower both patients and healthcare providers, informing the course of treatment through data-driven interventions while collecting data in real-time and potentially reducing the number of patient office visits needed. In particular, socially assistive robots (SARs), as a DTx tool, can be a beneficial asset to DH systems since data gathered from sensors onboard the robot can help identify in-home behaviors, activity patterns, and health status of patients remotely. Furthermore, linking the robotic sensor data to other DH system components, and enabling SAR to function as part of an Internet of Things (IoT) ecosystem, can create a broader picture of patient health outcomes. The main challenge with DTx, and DH systems in general, is that the sheer volume and limited oversight of different DH systems and DTxs is hindering validation efforts (from technical, clinical, system, and privacy standpoints) and consequently slowing widespread adoption of these treatment tools.

## 1. Introduction

Digital Health (DH) is a promising concept that could aid in delivering and managing care (1). DH systems traditionally include a blend of various technologies (i.e., wearables, smartphone apps), artificial intelligence (AI), and physiological biomarkers (2) demonstrating the potential to provide support for individuals with various health conditions. Eliciting and sustaining positive behavioral change is one of the main goals of healthcare practitioners as well as one of the main advantages of DH (3). In particular, changing traditional approaches to managing mental health disorders by introducing technology-based resources (i.e., mobile phones, computers, robots) may be a game changer in diagnosing and treating such conditions (4). More broadly, DH systems enable users of technology (both patients and health providers) to have deeper insights into underlying conditions that are more difficult to diagnose/manage via standardized behavioral observations in clinical settings. That approach allows us to think “outside of the box” when considering how to manage various health conditions, which is likewise important to maximizing the benefit of DH systems. For example, it has been reported that observing typing dynamics by using smartphone accelerometer data can help track and predict fluctuations in symptoms of mental health disorders beyond the clinic walls and map *real-world* bipolar disorder symptom trajectories in ways we could not before (5). Clearly, there is much potential for the future use of DH.

Nevertheless, in these early days of the DH era, mechanisms to validate the quality and benefits of these systems are still not rigorous enough. In this paper, we give an overview of current DH systems and discuss ongoing challenges, such as validation, that are slowing adoption of such technology in real-world practice. More specifically, the overarching aim of this paper is to introduce Socially Assistive Robots (SARs) as potential digital therapeutic tools (DTx), and as a beneficial asset to DH systems more broadly. Unfortunately, a very limited body of existing published research focuses on the utilization of SARs as DTx and in DH systems. As such, this paper should be treated as an introduction to the topic for researchers unfamiliar with SARs and DTx, summarizing some vital research in the field with the aim of stimulating more research in the future.

To provide such an introduction, Google Scholar was used to locate peer-reviewed journal articles from over the past decade. Keywords used in this search were: Digital Health, Digital Therapeutics, Socially Assistive Robots, Treatment, Clinical Setting, In-The-Wild, Internet of Things, Therapeutic Support, Behavior Change, and Machine Learning. The primary criteria for selecting articles was if it contained information on both SARs and DTx, which resulted in *only* 26 articles. After first defining DTx for readers, we summarize that research below.

## 2. Digital therapeutics

Goldsack et al. (6) define DH systems as any technology that purposefully engages consumers and captures, stores, or transmits health data while supporting life science and clinical operations. DH is a rather broad concept, and various technologies, platforms, or systems can fit within that definition. Digital therapeutics (DTx), on the other hand, are a much more specific set of *technology-enabled interventions* within the broader DH sphere that are intended to produce a measurable therapeutic effect. There are three major requirements for an intervention to be considered a DTx. In short, it needs to be: (1) an actual treatment intervention (not just “information”), (2) evidence-based, and (3) utilize high-quality software/hardware to prevent, manage or treat medical conditions (7). When fitting all three requirements, a DTx tool can theoretically be an ideal tool to empower both patients and healthcare providers by informing the course of treatment through data-driven interventions, while collecting data in real-time and reducing the number of patient office visits needed (8, 9). Depending on the course of treatment, a DTx can be used as a standalone approach (monotherapy) or as an addition to the already existing or prescribed treatments (adjunctive therapy).

According to the Digital Therapeutics Alliance (10), a leading international organization on digital therapeutics, all products claiming to be a DTx must adhere to ten foundational principles:

1) Prevent, manage, or treat a medical disorder or disease.
2) Produce a medical intervention that is driven by software.
3) Incorporate design, manufacture, and quality best practices.
4) Engage end users in product development and usability processes.
5) Incorporate patient privacy and security protections.
6) Apply product deployment, management, and maintenance best practices.
7) Publish trial results inclusive of clinically meaningful outcomes in peer-reviewed journals.
8) Have results reviewed and cleared or approved by regulatory bodies as required to support product claims of risk, efficacy, and intended use.
9) Make claims appropriate to clinical validation and regulatory status.
10) Collect, analyze, and apply real-world evidence and/or product performance data.

Interestingly DTx has been compared to traditional medications, paralleling the significance of this emerging treatment approach to lifesaving chemical compounds (7, 11). Recchia et al. (11) emphasize the similarity of DTx concepts to the ones of traditional medications stating that both possess active ingredients (i.e., a component dedicated to producing certain clinical outcomes) and excipients (i.e., an “inactive ingredient” that serves to ease/aid delivery) which work together to ensure prolonged use of therapy and acceptable patient experience while undergoing that treatment (12). Where DTx differs from traditional medications is the nature of the (digital) active ingredient and (digital) excipient. While still being responsible for the clinical outcome effect, the digital “active ingredient” is an algorithm instead of a chemical compound. The digital excipient, in this case, includes any type of virtual support or reward mechanism, that help propel the use of DTx and maximize the influence of the digital algorithm (11). More specifically to the focus of this paper, researchers have found phenomenon similar to that described above, such as robotic therapeutic interventions having the same effectiveness as medications for clinically depressed patients (13). We return to the topic of robots as DTx tools later in this paper.

## 3. Challenges with DTx

One of the main problems of DH, and consequently DTx, is associated with the increasing number of systems, software, and technology platforms advertised as DH or DTx. In 2017, funding allocated to the digital health sector was close to $6 billion, with more than 200 mobile health apps added daily to application acquisition portals (1). In 2021, the amount invested in DH systems around the world hit an all-time high of $57.2 billion, with US-based DH startups accounting for $29.1B across 729 deals, nearly doubling 2020’s US investment record of $14.9 billion (2). With the increasing oversaturation of DH systems and DTx tools on the market, it is becoming more and more challenging to validate their effectiveness. As mentioned above, to be broadly used, DH systems need to go through *rigorous* testing and be supported with validated *evidence* of their effectiveness (particularly in comparison to existing DH systems, i.e., comparative effectiveness). However, the sheer volume and limited oversight of different DH systems and DTx tools is hindering such validation efforts (whether from a technical, clinical, system, and/or privacy standpoint) and consequently slowing the widespread adoption of these treatments tools.

To compound the above problems, there are a growing number of DTx tools that integrate some form of AI into them. That often entails machine learning (ML) or deep learning (DL) techniques that seek to enhance DTx effectiveness by incorporating predictive models. However, enabling such ML/DL capabilities typically requires large amounts of relatively high-quality data, which is not always easy to access in the healthcare domain, particularly for specific diseases (14). There is also the issue of “explainable AI”, which are AI-enhanced tools that not only make predictions but can also explain to the user why such predictions were made for a particular case (15, 16). Explainable AI is of particular need in healthcare, where clinicians need to be able to communicate to patients the reasons for certain treatment recommendations in order to ensure treatment adherence (or potential medical liability reasons). However explainable AI is still a relatively new concept and remains an unsolved problem for DTx, and beyond (17).

Other concerns surrounding the implementation of such DTx tools are the privacy and security of the health information collected by AI. Those entail both concerns over the security of the storage, such as precautions that need to be taken to protect medical data, as well as the ability of the individual users to understand if and how they can maintain their privacy while still integrating these new technologies into their care plans (18).

Last but not least, a uniform approach to reimbursement for DTx tools is needed. It has been roughly five years since the US Food and Drug Administration (FDA) approved the first DTx tool for market, and to this date, the Centers for Medicaid and Medicaid Services (CMS) has not established clear guidelines on reimbursement for DTx (19). Yet, when robotic therapeutic interventions are deployed with individuals diagnosed with intellectual and developmental disabilities (IDD) as a recreation therapy intervention on the Medicaid Waiver program within some states, it is reimbursable. The problem with this reimbursable platform is that it is only reimbursable if coded specifically under recreation therapy and when the clinician is knowledgeable and trained on how to deploy DTx in that particular therapeutic format. When recreation therapists are introduced to forms of DTx, they feel it is an appropriate and useful intervention that has multiple benefits for the clients they work with (20). The challenge (or roadblock perhaps) is there are very few states who have recreation therapy as reimbursable therapy under the Medicaid Waiver making it difficult for individuals to access appropriate and affordable healthcare. In short, conflicts between federal and state policies are hindering the growth of DTx adoption.

## 4. Socially assistive robots as active ingredients in DTx

SARs have been traditionally used within the human-robot interaction (HRI) field to coach, motivate, and influence behavior change in people (21) and have been shown to be effective in achieving positive health outcomes with various populations. Previous research has shown that SARs help in the development of social skills, reducing loneliness and effects of social isolation, and providing general emotional support and companionship across the lifespan as a form of DTx (13, 22, 23). Beyond the aforementioned benefits, SARs can be a beneficial asset to DH systems more broadly, since data gathered from sensors onboard the robot can help identify the in-home behaviors, activity patterns, and health status of patients remotely. Furthermore, linking the robotic sensor data to other DH system components can create a broader picture of patient health outcomes (24).

To that latter point, SARs can also function as part of an Internet of Things (IoT) ecosystem, which utilizes a network of interconnected devices in people’s homes to holistically monitor and understand their home environment and daily lifestyle, beyond the scope of any individual device’s sensor capabilities. This joint IoT data collection approach can also help to better understand culture-specific behaviors performed by patients and identify interactions that patients have with SARs that produce positive health outcomes. Perceptions and use patterns of SARs, additionally, are known to vary significantly across cultures (25–27) and, as a result, lead to differences in how people utilize SARs to help them address health-related issues. Exploring culturally variable modes of use that bring out the beneficial effects of SARs in naturalistic home environments, in the long run, can help develop more culturally-sensitive designs and implementation of SARs as a form of ecological momentary intervention (EMI). EMIs are defined as individualized treatments that can be provided remotely in real-time via technology (28), Shiffman et al. (29), thus fitting into the concept of DTx and transforming an IoT-enabled SAR companion into an easily accessible “active ingredient” of a DTx tool. Such SAR-based EMI typically take the form of therapeutic interactive behaviors the SAR engages in with the person. Furthermore, EMI can take advantage of IoT data in order to help inform person-centered, individualized treatments to address the onset or progression of a certain condition, particularly if it is associated with environmental triggers related to a person’s living situation (e.g., stressors in autism or dementia).

Home environments are traditionally very difficult to access and unobtrusively collect data in yet are a focal space in patient’s lives that can provide health professionals valuable insight, beyond the clinic walls, into the underlying causes of health-related issues. SARs deployed in patients’ homes thus provide a unique way of interactively collecting environment and activity data. Developing ML/DL models could enable real-time assessment of patients’ interactions with the SAR and establish culture-specific predictive models of the effect the DTx has on patients’ health conditions. The authors of this paper have already deployed a pilot version of such a DH system (27, 30) using an adjunctive sensor device attached to a commercially available robot (Hasbro Joy for All). The resulting data and machine learning models are being used to further inform the design of Therabot (31), a robotic companion pet in the form factor of a dog. The Therabot platform exhibits awareness of its environment by responding to environmental factors, such as sound and touch. It is embedded within a DH system that increases the robot’s therapeutic value by identifying which particular sensors are useful for tailoring its behaviors to users’ needs, and linking that with smartphone and clinical data. The next steps that our research team intends to embark on are aligned with initiatives that DH and DTx research community as whole needs to undertake: (1) expanding DTx studies to provide more robust and generalizable results that enhance validation efforts, (2) identifying more potential IOT-enabled DTx “active ingredients”, and (3) integrating those active ingredients with a broad range of potential excipients (e.g., mobile phones, wearables, smart home devices, virtual reality), in order to enable easier access to beneficial therapies for patients across diverse cultural and geographic locales.

We should note briefly that not all the computational processing needs to occur directly onboard the devices (e.g., robot) for DTx applications. By means of cloud-based ML running on the backend of an IoT system, models can be created that inform healthcare practitioners about approaches to treatment individualization using real-time assessment of patients’ interaction with the SARs, without relying on expensive local hardware (32). Moreover, working in collaboration with health professionals, such approaches can be identified that integrate with the overall DH system while also fitting into existing clinical workflows. In this way, SARs can be thought of as *both* excipient and active ingredients in a DTx context, aiding medical practitioners alongside existing in-clinic patient tracking systems in order to more thoroughly monitor the changing behavioral, cognitive, and socio-emotional needs of patients. From a “data science” perspective, one might say that we are providing *actionable information* to clinicians and the overall ecosystem of care with regards to fluctuations in their patient’s health conditions over time. Every action the patient or clinician does causes an effect, which should then impact future actions. DTx tools such as SARs described here can serve as that “link” between actions now and future actions.

However, to execute such a “link” successfully the DH system needs to include different data sampling strategies and stimulus designs to collect relevant information from patients at appropriate times (24), all while the robotic sensors and connected IoT devices are collecting interaction data between the SAR and patient as well as the general home environment. By doing all those simultaneously, the system could provide enough robust data for researchers to understand in detail differences in context-specific robotic and human behaviors performed, the health status of the patients, and the home environment, while connecting those to the effects of a SAR-based DTx in real-time. In other words, a *robust* measure of effectiveness.

## 5. Discussion

In summary, acknowledging that investments in DH systems around the world in 2021 were close to $60 billion, there is no denying that AI-driven DTx tools hold vast potential to revolutionize the healthcare industry. SARs represent one such DTx tool that can exist as part of a broader DH system, offering a unique opportunity to collect data in real-time and track behavior changes, activity patterns, and general health status remotely. Furthermore, tying the robotic sensor data to other DH system components can create a broader picture of patient health outcomes while functioning as part of an IoT ecosystem, to holistically monitor and understand how a patient’s home environment and daily lifestyle impact health outcomes.

Nevertheless, there are multiple challenges that represent limitations currently, which need to be addressed before these systems fulfill their potential of improving patient outcomes and reducing healthcare costs. The main challenge is to successfully validate the effectiveness of the systems that are continuously emerging, and to do so in a replicable rigorous way. Unfortunately, many DTx tools currently on the market lack such rigorous validation, or any sort of *comparative effectiveness* research regarding their utility compared to competing DTx tools. The lack of effective validation approaches is the main reason for the slow adoption of these systems and treatments in real-world settings. ML/DL capabilities of DTx tools could help speed up the validation processes, by introducing a “data science” perspective that clearly links the decisions/actions of patients and providers to their effects. Nevertheless, collecting a sufficient amount of high-quality data to enable ML/DL capabilities is a challenge in itself. The authors of this paper urge future creators of DH systems and DTx tools to plan on more rigorous research steps as a follow-up to initial feasibility studies. Feasibility alone is not enough to ensure user adoption within a healthcare context. Those steps should entail expanding DTx studies to provide more robust and generalizable results towards validation, identifying more potential IOT-enabled DTx “active ingredients”, and integrating them with a broader range of potential excipients (e.g., mobile phones, wearables, smart home devices, virtual reality). By doing so, we can ensure faster adoption of these tools into real-world clinical practice, across a range of cultural and geographic locales.

There are, of course, other challenges that need to be addressed, such as privacy concerns and reimbursement issues that we mention in Section 3 of this paper. For instance, implementing systems to regularly assess the stability of digital health technologies and check who is accessing the data collected by such technologies is crucial (33), while providers some direct control over that access (18). For SARs as DTx tools in particular, expanding access through insurance reimbursement will likely necessitate training of therapists to use the tools and collecting more real-world outcomes data to persuade payors of its efficacy, otherwise patients will have to bear the brunt of the costs themselves. Regardless, such concerns as privacy and affordability are also likely to impact the speed of adoption, and cannot be ignored.
